# Annotations

**Published:** 1906-12-22

**Authors:** 


### ANNOTATIONS.

### The Election of Direct Representatives.

The recent election of representatives to the General Medical Council, whatever other criticism may be passed on it, has at least been characterised by a great deal of interest and a great deal of effort. All this is to the good, as showing an increasing appreciation of the necessity for solidarity in the ranks of the profession. Unfortunately, as the poll shows, it may still be urged that great numbers of the profession take little or no interest in the public affairs or relationships of their calling. With some this is mere indifference, whilst others—superior souls, doubtless—proclaim scornfully their entire lack of concern with "medical politics." Only too often, however, it is from the very persons who thus ostentatiously stand aside that are heard hostile comments on the corporate action of the profession. They appear to forget that the misdirection of affairs, granted it occurs, is due, on their own showing, to their own wilful and deliberate abstinence. However, our business now is not criticism, but congratulation. It is wholly to the good that candidates should be interviewed, committees formed, circulars issued, and addresses delivered. If nothing else is accomplished by the institution of all this election apparatus, it at least promotes interest and encourages the development of a corporate spirit. We have never hesitated to draw attention to the fact that the General Medical Council has but a limited opportunity in reference to many of the reforms concerning which the general body of the profession is anxious. But it is right and proper that direct representatives of the profession should be heard in this supreme executive body, and we fully recognise that the value of such influence, both direct and indirect, is considerable.

### Annual Dinner of the London Polyclinic.

The eighth annual dinner of the Medical Graduates' College and Polyclinic was given in the Trocadero Restaurant on the evening of December 12. There was a very large attendance of the members and their friends, the numbers present being in excess of any previous year and counting upwards of 200. The chair was occupied by Professor Clifford Allbutt, M.D., LL.D., F.R.S. In proposing the toast of "The Polyclinic," the Chairman referred to his early association with the clinical teaching of the College, and to his interest as a Professor of Medicine in the organisation of post-graduation studies. Whilst there are clear and distinct indications that to an ever-increasing degree the problem of medicine must be realised as the prevention of disease, it was manifest that the successful solution of this problem was yet a long way ahead. Again, though laboratory work was of the highest value and was able to arrange facts with a view to the announcement of general principles, the translation of such principles into successful practice at the bedside was hindered by many practical obstacles and complexities from which the laboratory worker was altogether free. Hence the clinical observer, having his patient before him, was not helped by methods of prophylaxis nor, at least in many instances, by the voices from the laboratory. He remained charged with responsibility towards both the individual and the community. In this responsibility he needed help and encouragement, and such influences were to be obtained in institutions like the Polyclinic, which was mainly devoted to the actual problems presented to the practitioner in his daily work. For this reason alone, and apart from others, the Polyclinic was worthy of the support of the profession, and it was satisfactory to know that its function and value were receiving increasing appreciation. The Medical Superintendent, Captain Hayward Pinch, received a very cordial welcome and was heartily cheered by the members present.

### Hastings as a Winter Resort.

Increasing railway facilities tempt people nowadays to go farther afield than of old, even in the winter, and the Christmas holidays see every year more and more people wending their way southward. For invalids, a stay in some milder climate may mean all the difference between well-being and wretchedness. But it is strange, and especially strange of those whose health is delicate, that they should so often seek their warmer climate far from home. Continental resorts may be pleasant enough in the summer, but when days are short and nights chilly it is a good thing to be within reach of a cosy English fireside and among people of one's own nationality who are in sympathy with one's ideas. Of course, when we go from home in the winter it is in search of the sun. But the sun shines on our own shores as well as elsewhere. Those who have visited Hastings in the summer might reasonably complain of a superabundance of sunshine; but this is all the more encouraging when one thinks of the town as a winter abiding place. In December the sunshine is more than welcome, and strolling along the splendid esplanade, or resting on one of the seats whose glass sides protect from all but the warmest breezes, no one need envy friends who have taken a long and fatiguing journey across Europe for the sake of basking by the Mediterranean. There are other advantages. There need be no breaking of home ties when those members of the family who need the change are within easy reach of the others, so that a daily journey from town is not too much for the head of the house, and a week-end trip merely a pleasant change. Moreover, there is the important fact that in an English town we have the assurance of decent sanitation, not only in the hotel or lodging, but in the whole town. Hastings can boast of a death-rate so small that it might arouse the envy of other places. The death-rate for last quarter was only 12.8 per 1,000 of population, which is far below the average for the country as a whole. And it must be remembered that many old and delicate people make their home in Hastings, going there only after their years of health and vigour are past, and therefore abnormally increasing the proportion of deaths. That, in spite of this element in the population, the rate continues so low is a striking proof of the healthiness of sunny, yet airy, Hastings.

